# The Influence of Different Protocols on the Application of the Dithiothreitol Assay in Determining the Oxidative Potential of Ambient Particles

**DOI:** 10.3390/toxics13020113

**Published:** 2025-01-30

**Authors:** Maja Jovanović, Marija Živković, Bojana Petrović, Saima Iram, Milena Jovašević-Stojanović, Svetlana Stevanović

**Affiliations:** 1 VIDIS Centre, Vinca Institute of Nuclear Sciences—National Institute of the Republic of Serbia, University of Belgrade, 11 000 Belgrade, Serbia; marijaz@vin.bg.ac.rs (M.Ž.); bojana.petrovic@vin.bg.ac.rs (B.P.); mjovst@vin.bg.ac.rs (M.J.-S.); 2 School of Engineering, Deakin University, Burwood, VIC 3216, Australia; s223015208@deakin.edu.au (S.I.); svetlana.stevanovic@deakin.edu.au (S.S.)

**Keywords:** oxidative potential, dithiothreitol assay, particulate matter

## Abstract

Environmental particulate matter (PM) exposure has been widely recognized for its significant adverse effects on human health. Monitoring PM levels is one of the essential parameters of air quality assessment. However, PM mass concentration alone does not sufficiently explain its toxicological impacts and effects on health. This study highlights the importance of oxidative potential (OP) as a promising metric for evaluating PM toxicity. It focuses on standardizing the dithiothreitol (DTT) assay as a tool for OP measurement. In order to investigate the impact of various extraction techniques, reagent concentrations, and assay conditions, four previously established protocols were tested without modification, while a novel protocol was introduced based on an extensive literature review. Results revealed strong positive correlations between the new and most established protocols. These findings highlight the significance of the new protocol in advancing the development of standardized methodologies for applying the DTT assay and demonstrating its reliability and relevance. While developing a standardized DTT assay involves addressing numerous parameters—from filter extraction to assay application—this research provides a solid base for achieving consistency in OP measurements and overcoming this critical issue.

## 1. Introduction

Increased exposure to ambient particulate matter (PM), particularly smaller particles (less than 2.5 µm), has been extensively documented to have profound adverse health impacts, inducing various inflammatory processes and contributing to the incidence of cardiovascular and respiratory diseases [1,2,3,4,5]. Furthermore, numerous studies conducted on human cell lines and animal models have unequivocally demonstrated the carcinogenic and mutagenic effects of particulate matter [6,7]. Given the detrimental impacts of exposure to particulate matter, assessing PM levels is a fundamental aspect of comprehensive air quality monitoring initiatives. While mass concentration serves as the prevailing metric for PM regulation, recent research underscores the inadequacy of this measure alone, emphasizing the critical role of PM’s complex chemical composition, particle number concentration, surface area, and other intrinsic properties in eliciting hazardous reactions [8]. On the other hand, merely assessing PM mass is insufficient to fully comprehend its potential impacts on human health and the environment, necessitating a more comprehensive understanding of particle composition. While the precise mechanisms underlying the detrimental effects of particles on human health remain elusive, researchers have proposed several theories to elucidate this phenomenon. Among these theories, the generation of reactive oxygen species (ROS) and subsequent oxidative stress in biological systems stands out as a prominent hypothesis. Oxidative stress, triggered by the production of ROS, is believed to play a pivotal role in the pathophysiological processes associated with particulate matter exposure, contributing to the increased risk of developing various health problems [9]. Consequently, the oxidative potential of PM, which quantifies its capacity to oxidize target molecules and induce ROS production in a cell-free environment, holds promise as a valuable descriptor of its toxicological properties. Notably, the correlation between PM mass concentration and oxidative potential can vary significantly, underscoring the importance of discerning particle composition in assessing toxicity. Moreover, the sources contributing to PM mass concentration often diverge from those driving oxidative potential, highlighting the limitations of mitigation strategies solely focused on reducing PM concentration [10]. Therefore, considering that oxidative potential is associated with health effects, it would be advisable to implement it in a reduction strategy. The importance of measuring the oxidative potential as an important parameter in air quality monitoring was additionally recognized by the European Commission. Namely, in the last directive published in October 2024, proposed by the European Union, in addition to the standard parameters that are monitored in regular monitoring, OP of PM was added as a new air pollutant that should be included in the air quality monitoring assessment process [11].

The scientific literature abounds with a variety of acellular (cell-free) assays designed to evaluate the oxidative potential of particulate matter, such as ascorbic acid (AA), reduced glutathione (GSH), dichlorofluorescein diacetate (DCFH), dithiothreitol (DTT), electron spin resonance (EPR), etc. [12,13,14,15,16]. Among these assays, the dithiothreitol assay stands out as one of the most widely utilized methodologies, tracing its origins back to 2002 when it was first introduced by Kumagai et al. [17]. DTT serves as a proxy for cellular reductants like NADH/NADPH, facilitating the simulation of interactions between physiological reductants and aerosol components via chemical analysis [18]. Aerosol components may engage in direct reactions with antioxidants or facilitate electron transfer from antioxidants to dissolved oxygen, resulting in antioxidant depletion and, in the next phase, both antioxidant depletion and ROS generation. During the application of this assay, DTT represents a physiological reductant, and monitoring its consumption over time under conditions of excess DTT yields a proportional measure of redox-active species concentration present in PM [19]. Despite the availability of numerous acellular tests, the DTT assay has widespread adoption primarily due to its application simplicity and the affordability of the required chemicals. However, a significant obstacle to the widespread implementation of these assays, including DTT, lies in the absence of standardized procedures, which complicates comparing results across different studies. In many scientific publications, researchers predominantly rely on either the protocol proposed by Cho et al. [20]. or a modified version developed a few years later by Li et al. [21]. Although these protocols serve as the basis for many studies, authors often introduce additional modifications, ranging from extraction process alterations to reagent concentrations, incubation times, or even the omission of specific steps. To ensure the accurate application of the DTT assay to real particulate matter samples, whose composition can significantly vary depending on the source, it is crucial to address potential errors arising from different extraction procedures and methodological inconsistencies. By refining and standardizing the DTT assay protocol based on insights gleaned from the available scientific literature, researchers can enhance the reliability and reproducibility of oxidative potential measurements, thereby facilitating meaningful comparisons across studies and advancing our understanding of the health implications associated with particulate matter exposure.

In light of the considerations mentioned above, the principal objective of this study is to conduct a comparative analysis of five distinct protocols utilized in applying the DTT assay. By scrutinizing how variations in experimental conditions and factors influence the final outcomes, this research aims to provide valuable insights into the efficacy and reliability of different methodologies. Additionally, with the aim of establishing a standardized procedure for the application of the DTT assay, this paper introduces a novel modified protocol that can serve as a starting point in overcoming this issue. This innovative approach is poised to streamline future research endeavors, offering researchers a comprehensive framework for conducting oxidative potential assessments with heightened precision and consistency.

## 2. Materials and Methods

### 2.1. Site Description and PM_2.5_ Sampling

The PM_2.5_ samples were collected at the automatic station Ada Marina, Belgrade (44.790284, 20.417169), which belongs to the network of automatic measuring stations for air quality monitoring run by the Public Health Institute of Belgrade. This measuring station is located next to the river island of Ada Ciganlija, in the central zone of the Belgrade metropolitan area, 6.3 km away from the strong city center, surrounded by greenery. At the same time, the key roads (urban freeways) with high traffic intensity runs in the vicinity (approximately 100 m), which is why traffic is the most significant contributor to pollution. In order to cover a broader range of particle concentrations and examine the effect of mass on the response of the DTT assay, the PM_2.5_ samples were collected every fourth day from 6 June 2023 to 28 June 2023 and from 5 December 2023 to 31 December 2023. The referent sample pump for PM_2.5_ was put about 2 m from the ground, and the sampling collection lasted 24 h, from midnight one day to the same time the next day.

A low-volume sampler (LVS3, Sven Leckel, Berlin, Germany) equipped with a PM_2.5_ inlet head was employed to collect particles with a flow rate of 2.3 m^3^/h. Particles were collected on the 47 mm quartz fiber filters (Whatman QMA), equilibrated (48 h at 40% RH, 20 °C), and weighed with a microbalance (MYA 5.3Y, RADWAG, Radom, Poland) before and after the sampling. After sampling, filters were kept in Petri dishes, immediately wrapped in aluminum foil, and stored in the refrigerator at −20 °C until the OP analysis. In addition, four filter blanks were collected in order to check the quality of PM measurements.

### 2.2. Filter Extraction

For consistency across all experimental procedures in this study, a uniform filter area was employed for each of the five protocols under investigation. Utilizing a pre-cleaned cutter, the sampled filters and the blank filters underwent meticulous division into ten precisely equal circles. Each circle boasted a diameter of 0.9 mm, corresponding to a surface area of 0.64 cm^2^, with a total of two circles, equivalent to 1.28 cm^2^, utilized for each individual protocol. The subsequent steps of the experimental protocols, delineated in detail in the forthcoming section, entailed the application of ultrasound extraction techniques for varying durations or a combination of sonication, vortexing, and shaking, depending on the specific protocol being executed. Additionally, depending on the protocol, ultrapure deionized water (18.2 MΩ) or methanol (high purity grade > 99.9%, p.a.) was used as the solvent.

### 2.3. Different DTT Protocols

#### 2.3.1. Protocols 1 and 2

Protocol 1 (Simplified Cho) and Protocol 2 (Modified Li) were taken from the paper published by Lin and Yu, 2019 [22]. Both procedures are a modification of the original protocol for determining the oxidative potential using the DTT assay [20], and the second procedure was published a few years later [21]. The authors used 1.5 to 3.0 cm^2^ of filter sample for both protocols and extracted it in 4 mL of deionized water in an ultrasonic bath for 40 min. As the filter surface of 1.28 cm^2^ was used for the purpose of this work, the same volume of ultra-pure water was taken for extraction, as suggested by the authors of those modified procedures. After the extraction, each sample was filtrated using a syringe filter (0.45 µm PTFE).

Both protocols are actually the original procedures proposed by authors [6,7] with some modifications. Firstly, the phosphate buffer was treated with Chelex 100 resin instead of using 1 mM Ethylenediaminetetraacetic acid (EDTA) to remove trace metals. Secondly, for Protocol 1, the initial DTT concentration that Cho used (100 µM) is too high and above the linear response range of the subsequent absorbance measurement, so additional dilution was introduced in the penultimate step to reduce the concentration to 20 µM mm. For Protocol 2, the initial DTT concentration was 20 µM. Additionally, both protocols excluded the addition of HCl buffer and Trichloroacetic acid (TCA). All other steps are completely the same as in the original procedures. Table 1 illustrates the concentrations of reagents for all five protocols used in this work, while Figure S1 presents the flowchart with the individual steps of each protocol.

OP measurement for Protocol 1: 1 mL of potassium phosphate buffer (0.1 M, pH = 7.4) and PM extract samples (200 µL) were mixed and incubated at 37 °C. Following this, 50 µL of DTT (2.5 mM) was added to initiate the oxidation reaction. At intervals of 0, 10, 20, and 30 min, 50 µL of 5,5-dithio-bis-(2-nitrobenzoic acid)—DTNB (10 mM) was added to the reaction mixture, allowing it to form TNB. In the next step, 0.2 mL of the mixture was taken out and transferred into another vial previously poured with 0.8 mL of phosphate buffer, and the absorbance spectrum was measured at 412 nm using a UV–VIS spectrometer.

OP measurement for Protocol 2: 1 mL of potassium phosphate buffer (0.1 M, pH = 7.4) and PM extract (200 µL) were mixed and incubated at 37 °C. Following this, 50 µL of DTT (0.5 mM) was added to initiate the oxidation reaction. At intervals of every 10 min, 50 µL of DTNB (10 mM) was added to the reaction mixture, shaken well, and the absorbance spectrum was immediately measured at 412 nm using a UV–VIS spectrometer.

#### 2.3.2. Protocol 3

Protocol 3 was taken from the paper published by Wang and coauthors [23]. In this paper, the authors used a 16 mm diameter circular filter punch for winter samples in this protocol. Each filter sample was immersed in 10 mL of deionized water and extracted in an ultrasonic cleaner for 30 min. Since two circular filter punches (each with a 0.9 mm diameter) were used in this work, it was decided to apply the same amount of water. After the extraction procedure, the samples were filtrated through a 0.45 µm syringe filter. As in the case of the previous protocol, the phosphate buffer was treated with Chelex resin, and Tris HCl buffer and 10% TCA were added during the OP analysis. As can be seen from Table 1, the initial concentration of DTT was 100 µM.

OP measurement for Protocol 3: 4.55 mL of potassium phosphate buffer (0.1 M, pH = 7.4, treated with Chelex 100 resin) and PM extract samples (1.75 mL) were mixed and incubated at 37 °C for 5 min. Following this, 0.7 mL of DTT (1 mM) was added to initiate the oxidation reaction. At intervals of 0, 10, 20, and 30 min, aliquots were transferred to tubes containing TCA to stop the reaction. After that, 2mL of Tris-Base (0.4 M Tris with 20 mM EDTA, pH = 8.9) and 50 µL of DTNB (10 mM) were then added, allowing DTT to react with DTNB to form TNB. After 5 min incubation at room temperature, the absorbance spectrum was measured at 412 nm using a UV–VIS spectrometer.

#### 2.3.3. Protocol 4

Protocol 4 was taken from the paper published by Perrone and coauthors [24]. OP analysis was carried out on filters with a diameter of 2.5 cm (approximately 0.2–3.8 mg of PM), which were immersed in 4 mL of methanol and treated in an ultrasonic bath for 10 min. Subsequently, the samples were filtered through a syringe filter (0.45 µm); the methanol was evaporated in a stream of nitrogen to dryness and then dissolved in 1.6 mL of 0.1 M phosphate buffer. The same extraction procedure was applied in this work, with a slightly smaller filter area (two filter punches of 0.9 mm each). In addition, the steps in which it was added Tris HCl buffer and 10% TCA were excluded. In this protocol, the initial DTT concentration was 19 µM.

OP measurement for Protocol 4: the standard DTT procedure involved adding 750 µL of 0.1M phosphate buffer (pH = 7.2) and 250 µL of 0.1 mM DTT to a 4 mL vial, which was continuously shaken on a shaker at 37 °C. At the time zero, 300 µL of PM extract was introduced into the mixture, followed by the addition of 10 µL of 10.0 mM DTNB in phosphate buffer solution at predetermined intervals. For every PM sample, five vials were prepared, with DTNB added at intervals of 0, 5, 10, 15, or 20 min. The rate of DTT loss (µM min^−1^) was calculated based on the linear regression of DTT concentration over time (0–20 min) from these five data points. Additionally, the blank comprised 300 µL of 0.1 M phosphate buffer solution devoid of any added redox-active species.

#### 2.3.4. Protocol 5—This Work

Protocol 5 is the modified version of the two protocols presented in the paper by Lin and Yu, 2019 [22]. Following an extensive review of existing literature, the authors of this study present a novel protocol aimed at addressing the current challenges associated with the implementation of the DTT assay. To achieve this objective, as previously outlined, the authors employed two filter punches with a diameter of 0.9 mm submerged in 3 mL of methanol. Subsequently, the samples underwent a series of treatment steps, including 10 min of sonication, followed by 10 min of vortexing at 800 rpm, and a 1 h rotation at 120 rpm. As in the previous protocol, methanol was evaporated in a stream of nitrogen. Each sample was dissolved in 2 mL of deionized water and stored in a freezer until further analysis.

OP measurement for Protocol 5: for the measurement of oxidative potential, a mixture (A) was prepared consisting of 1 mL of 0.1 M phosphate buffer (pH = 7.4, treated with Chelex resin), 200 µL of PM suspension, and 50 µL of 1 mM DTT. Following incubation at 37 °C for varying durations (0, 10, 20, 30, and 40 min), aliquots of 200 µL from mixture A were transferred to separate Eppendorf tubes preloaded with mixture B, comprising 800 µL of phosphate buffer and 50 µL of 5 mM DTNB. Subsequently, the absorbance of the yellow TNB formed was measured at 412 nm using a spectrophotometer (Lambda 35 UV-Vis Spectrometer, Perkin Elmer, Inc., Waltham, MA, USA). Prior to sample analysis, the analytical device underwent calibration using a DTT calibration curve spanning a concentration range of 0–100 μM.

To mitigate the sensitivity of both DTT and DTNB to light exposure, all protocols were conducted within a darkened hood with ambient room lights turned off. Additionally, reagents were protected from light by wrapping them in aluminum foil and were maintained on ice throughout the analyses to ensure their stability and integrity.

### 2.4. Quality Assurance and Quality Control

Field blank samples were collected to assess potential background contamination during sampling, transport, and storage. A blank sample was analyzed for every protocol. The limit of quantification (LOQ) and the limit of detection (LOD) were determined using the standard deviation of the slope (μM DTT min^−1^) of the blank filters analyzed (10 and 3 times the standard deviation of the 5 replicates, respectively). The LOQ ranged from 0.024 to 0.165 μM DTT, and the LOD ranged from 0.080 to 0.550 μM DTT min^−1^. Data were adjusted for blank values and method recoveries.

Before measuring absorbance in the samples for each protocol, the analytical device underwent careful calibration to ensure accuracy and consistency. This calibration was conducted using a DTT calibration curve across a concentration range of 0 to 100 µM, with R² values for the calibration curves exceeding 0.98 for all protocols, which provided a reliable baseline for comparing results. For each protocol, varying concentrations of DTNB were prepared and tailored to the specific conditions to achieve optimal titration. The formation of TNB was subsequently measured at a wavelength of 412 nm.

## 3. Results

After applying all five protocols to determine the OP^DTT^ of PM_2.5_ on selected samples covering the summer and winter periods, descriptive indicators for each protocol are listed in Table 2. The OP^DTT^ values were determined and then calculated based on the total volume of air sampled (nmol/min/m^3^), providing a more precise measure of pollutant concentration in relation to actual air intake. This approach ensures that OP values directly reflect the real-world inhalation exposure, capturing the oxidative stress that individuals may encounter in polluted areas. Based on the obtained results, it can be observed that Protocol III showed the highest mean (1.084) value calculated for all samples, and also for summer (0.498) and winter (1.671) samples. In contrast, Protocol IV showed the lowest mean (0.045) value calculated for all samples set, as well as for summer (0.044) and winter (0.046). Comparing the standard deviation, Protocol III had the highest standard deviation (1.153). Protocol II and Protocol V also had higher standard deviations, suggesting more variability in their measurements. Protocol III recorded the highest maximum value (4.080), while Protocol IV recorded the lowest minimum value (0.013) and generally had the lowest values among the protocols. By comparing the maximum and minimum values, it was observed that Protocol III had the widest range, while Protocol IV had a moderate range.

To thoroughly investigate the variability among the applied protocols, the coefficient of variation (CV) was calculated using the following equation: CV = ((Standard deviation)/Mean) * 100. In this study, the CV was calculated for all protocols, considering summer and winter samples separately. The highest values of the coefficient of variation were determined for Protocol III and IV, while the smallest CV was for Protocol II. These CV values indicated a broad distribution of all protocols.

**Figure 1:**
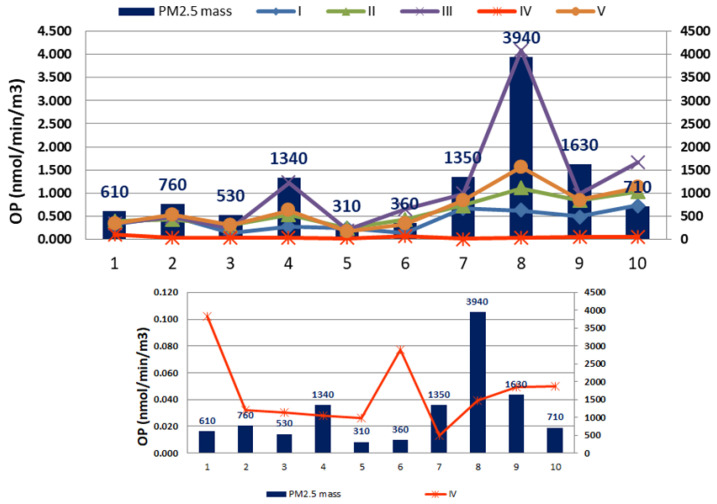
Variation of OP values for selected PM_2.5_ samples (the smaller graph represents only the variability of OP values for Protocol IV).

Figure 1 shows PM_2.5_ mass (µg) and OP^DTT^ values obtained using different protocols (I to V). As can be seen from the figure, the masses for the selected samples ranged from 310 to 3940 µg, while the OP values ranged from 0.013 to 4.080 nmol/min/m^3^. For summer samples (1–5), the masses ranged from 310 to 1340 µg, while for the winter samples (6–10), the measured masses were significantly higher, from 360 to 3940 µg. The measured OP values for summer and winter samples ranged from 0.026 to 1.226 nmol/min/m^3^ and from 0.013 to 4.080 nmol/min/m^3^, depending on the applied protocol. All protocols except the fourth had a similar trend with certain variations in the obtained values, especially for sample number 8, which had the highest PM mass. In general, the best agreement was found between Protocols II and V.

After conducting a thorough analysis of the descriptive indicators, the Shapiro–Wilk normality tests were applied to each assay. The analysis revealed that the results for four out of the five protocols did not have a normal distribution. Specifically, a normal distribution was observed only in the results obtained from Protocol I. The Kruskal–Wallis test was applied to examine the differences in the obtained median values and distribution between all five protocols. This test determined a statistically significant difference between the applied protocols. In order to more precisely determine which protocols have a significant difference in the obtained mean values, the Mann–Whitney U test was applied and p values are presented in Table 3. A statistically significant difference was found between Protocol IV and all others.

In order to examine the relationship between Protocols I, II, III, and V more deeply, Spearman’s correlation coefficients were determined (Table 4). A strong positive, statistically significant correlation (from 0. 648 to 0. 988) was established between Protocols I and II, I and III, I and V, and II and III and between II and V.

## 4. Discussion

This study aims to evaluate the distinctions between five different DTT protocols applied to obtain the OP of PM_2.5_. Starting with the extraction of the samples, it should be noted that for the first three protocols, water was used as a solvent and performed in an ultrasonic bath for 30 to 40 min. For Protocol IV, methanol was used as a solvent, and the extraction was performed for only 10 min in an ultrasonic bath. Finally, for Protocol V, multiple extraction techniques were used to ensure the most efficient removal of particles from the filter, and methanol was also used as the solvent. The phosphate buffer (0.1 M) used in all protocols was treated with Chelex resin before use, and for the needs of each protocol, it was treated with acid to adjust the pH value, which, depending on the protocol, varied from 7.2 to 7.4. As is already known, the solution’s acidity significantly influences the DTT assay, not only on the catalysis of redox reactions but also on the molar absorption coefficient of the yellow-colored TNB formed at the end. Accordingly, Li et al. [21] suggested 7.3 as the optimal pH value for the DTT assay. For the purposes of this study, a phosphate buffer whose values deviated very little from the optimal (±0.1) was used, so the buffer’s influence on the final result was excluded. Then, based on the concentrations of DTT and DTNB used in each of the protocols, molar ratios were calculated. The molar ratio for Protocols I, II, and IV was 1:4; for Protocol III, the ratio was much lower, 1:1.4, while for Protocol V, the ratio was slightly higher, 1:5. Further, it should be noted that only Protocol III contained a step in which TCA was added to quench the reaction, followed by the TRIS buffer containing EDTA.

Based on Table 2, it can be seen that Protocol III showed the highest OP values, indicating it generally measured higher DTT activity compared to the other protocols and also had the highest standard deviation, which suggests greater variability measured by this protocol. In contrast, Protocol IV showed the lowest mean and standard deviation, indicating it generally measured lower DTT activity. Very high CV values were calculated for Protocol III (approximately the same percent for both periods—85%) and Protocol IV (approximately 75% for summer and 50% for winter samples). Protocol I and V had similar CVs, around 45%, while the lowest CV was determined for Protocol II, around 30%. In general, one of the main reasons for the high variability around the mean value determined for protocols III and IV is the insufficient sample extracted for analysis. A critical limitation of these two protocols is their inability to facilitate the analysis of samples in duplicate or triplicate, which underscores a practical challenge in the experimental design. Insufficient sample volumes hinder the reliability and reproducibility of the results, highlighting the importance of considering the amount of sample extract when selecting a protocol for specific applications. However, it should be emphasized that the number of samples used in this study is an additional factor that could have influenced the result. A similar influence of the number of samples can also be observed with the other protocols where significant variability was obtained, but still almost twice as low compared to those two protocols. Still, the excellent agreement between Protocols I and V should also be highlighted.

Figure 1 shows that for individual samples, it is clearly seen that higher mass does not necessarily lead to higher OP values and vice versa. Samples with higher PM_2.5_ mass might show higher OP values. However, this is not always the case, considering the complexity of the chemical components involved in the composition of the particles. The best agreement for the tested protocols was determined between protocols II and V, then for protocols I and V, although slightly higher OP values were determined for protocol V. The reason for the higher values found for protocol V may be due to several factors, which will be explained below.

Based on the obtained p values for the Mann–Whitney U test, there is a statistically significant difference between Protocol IV and all others, showing significant differences between the obtained OP of PM_2.5_ values measured by the respective protocols. This indicates that using Protocol IV, consistently different (lower) OP values were obtained compared to other protocols. Protocol III, while showing a trend toward being different, did not reach statistical significance in these comparisons. These findings highlight that Protocol IV stands out as measuring distinctly different OP levels compared to the others, while the other protocols were more similar in their measurements. This result can be attributed to several factors. Firstly, starting from the assumption (which represents one of the limitations of this work) that the tested sample has a homogeneous distribution of particles on the surface of the filter, the extraction procedure for Protocol IV was performed in methanol using an ultrasonic bath for 10 min. It is generally known that sonication longer than 30 min forms ROS [25], which can lead to positive artifacts in determining OP by DTT. However, a brief sonication period of just 10 min, even when performed in methanol, is likely insufficient for the effective extraction of particulate matter. This limited duration may not provide adequate time for the sonication process to fully break down and release the particulate-bound compounds. Consequently, the efficiency of PM extraction is compromised, potentially leading to inaccurate measurement of the oxidative potential of the samples. Extending the sonication time could enhance the extraction process, ensuring a more thorough and representative analysis of the OP. Thus, optimizing the sonication duration is crucial for achieving reliable and consistent results in PM extraction. Secondly, a significant weakness of this protocol is reflected in the small volume of the extract. Namely, according to the protocol given by Perrone et al. [24], after extracting the sample in methanol that was evaporated to dryness in a stream of nitrogen, the sample was dissolved in 1.6 mL of phosphate buffer. Considering that each sample is measured at five different times, such a small volume is not enough to perform the protocol even in duplicate. This represents another significant limitation in obtaining a representative result. Finally, another significant difference between this protocol and the others is the time interval in which TNB formation was measured. The tested samples were analyzed by drawing every 5 min, which could affect the linearity and reliability of the obtained OP values. According to Visentin et al. [26], a linear relationship between data points is essential for achieving reproducible and reliable results. Consequently, a 10 min interval was selected between successive withdrawals.

On the other hand, the slightly higher values obtained for protocol V may be due to a more efficient extraction, considering that three different extraction techniques were used. The influence of different combinations of extraction procedures on OP values has not yet been sufficiently investigated. Although methanol has lower polarity than water, it was decided to use methanol as an extraction solvent in Protocol V. Compared to water, methanol allows better extraction of hydrophobic components that could affect the DTT response. Higher values of OP during extraction in methanol were also determined in previous studies [27,28,29], which was an additional reason for choosing this solvent.

As the comparison of mean values is not a sufficient indicator to determine differences between protocols, Spearman’s correlation coefficients were determined to examine the relationship between protocols more deeply. Protocols I, II, III, and V showed strong positive correlations with each other. For instance, Protocols II and V had correlations of 0.988 (significant at *p* < 0.01), and Protocols II and III had correlations of 0.939 (also *p* < 0.01). These high correlations suggest that these protocols are largely aligned in measuring similar aspects of PM_2.5_ oxidative potential, making them comparably reliable for related analyses. Protocol IV shows weak or near-zero correlations with the other protocols, particularly with Protocol I (−0.042) and Protocol III (0.091). However, the agreement obtained between the analyzed protocols represents only one aspect of understanding the lack of a standard procedure for applying the DTT assay. Although this paper included samples from two seasons, which covered a fairly wide range of particle masses, one of the limitations of this research is the small number of samples. Furthermore, chemical composition and meteorological conditions can significantly affect OP values regardless of the assay used for its determination. Additionally, comparing the obtained OP values from different locations can also significantly change this agreement. Further, in the indoor environment conditions, when the OP values are much lower, another aspect not covered by this study is that it can also affect the agreement of different protocols. For this reason, establishing a unique DTT protocol has a crucial role in determining the oxidative potential as one of the important parameters in assessing the adverse health effects of particles.

**Future work:** Although establishing a standard method for applying the DTT assay is a complex process that should include many parameters, starting from the selection of the sampling filter to the application of the assay itself, this work can serve as a reasonable basis for overcoming this issue. Future research should also focus on the choice of solvent and extraction method, which, in our opinion, should include several extraction techniques and on the final volume of the sample, which can have a decisive importance on the final OP value.

## 5. Conclusions

In this study, five different DTT protocols were applied to examine the influence of their differences, varying from the extraction procedure to the concentration of reagents used. Four protocols were taken from the literature, while the fifth protocol was proposed by the authors of this study after a detailed analysis of the existing literature. Based on the results, the lowest oxidative potential values were obtained using Protocol IV, whereas Protocol III yielded the highest values, indicating considerable variability between protocols. While all protocols exhibited relatively consistent trends, Protocol IV showed a significant deviation from the others, suggesting potential limitations in its repeatability, applicability, or sensitivity. Notably, all applied protocols and the novel protocol demonstrated a good correlation with each other except Protocol IV, further emphasizing the discrepancies associated with this one. This deviation from Protocol IV was probably a consequence of insufficient extraction, inadequate sample volume for applying this protocol in duplicate, and the five-minute withdrawal of samples from the reaction mixture. When setting up an adequate procedure for applying the DTT essay, these important parameters were overcome precisely through the novel protocol proposed in this paper. The novel protocol demonstrated consistent performance and reliability through rigorous validation processes, including assessments of detection and quantification limits, linearity, range, robustness, and selectivity. The meticulous optimization of factors such as extraction efficiency proved instrumental in achieving method robustness.

From our perspective, future research should prioritize establishing the optimal extraction procedure, incorporating multiple extraction techniques to identify the most effective and representative approach. Additionally, a crucial parameter in optimizing the protocol is determining the appropriate volume of the extract for analysis, as this can significantly impact the accuracy and reliability of the results. Addressing these aspects will be essential for enhancing the reproducibility and robustness of OP assessments.

## Figures and Tables

**Table 1:** Differences in the steps and concentrations used when applying the DTT assay.

Protocol	Extraction Procedure	Concentration of Phosphate Buffer, pH	DTT conc.	DTNB conc.	TCA	Tris HCl + EDTA	Initial DTT conc.	Final DTT conc.	Reference
1	40 min sonication (in water)	0.1 M, pH = 7.3, Chelex	2.5 mM	10 mM	/	/	100 µM	19.23 µM	[22]
2	40 min sonication (in water)	0.1 M, pH = 7.4, Chelex	0.5 mM	1 mM	/	/	20 µM	18.52 µM	[22]
3	30 min sonication (in water)	0.1 M, pH = 7.4, Chelex	1 mM	10 mM	10%	0.4 M Tris + 20 mM EDTA, pH = 8.9	100 µM	16.67 µM	[23]
4	10 min sonication (in methanol)	0.1 M, pH = 7.2, Chelex	0.1 mM	10 mM	/	/	19 µM	18.85 µM	[24]
5	10 min sonication, 10 min vortexing, and 1 h rotation (in methanol)	0.1 M, pH = 7.4, Chelex	1 mM	5 mM	/	/	40 µM	7.62 µM	This work

**Table 2:** Descriptive indicators of DTT activity (nmol/min/m^3^) of PM_2.5_ for five applied protocols.

		Protocol 1	Protocol 2	Protocol 3	Protocol 4	Protocol 5
**All** **samples**	Mean ± std	0.415 ± 0.220	0.606 ± 0.305	1.084 ± 1.153	0.045 ± 0.027	0.670 ± 0.431
	Range	0.137–0.732	0.247–1.107	0.199–4.080	0.013–0.102	0.177–1.557
**Summer**	Mean ± std	0.298 ± 0132	0.382 ± 0.111	0.498 ± 0.421	0.044 ± 0.033	0.398 ± 0.180
	Range	0.140–0.502	0.247–0.530	0.199–1.226	0.026–0.102	0.177–0.624
	CV (%)	44.49	29.19	84.66	75.05	45.20
**Winter**	Mean ± std	0.533 ± 0.238	0.831 ± 0.267	1.671 ± 1.398	0.046 ± 0.023	0.941 ± 0.449
	Range	0.137–0.732	0.436–1.107	0.649–4.080	0.013–0.077	0.328–1.557
	CV (%)	44.61	32.17	83.66	50.52	47.71

**Table 3:** *p* values of the Mann–Whitney U test for five selected protocols.

	I	II	III	IV	V
**I**	/	0.151	0.096	**0.0002**	0.131
**II**		/	0.496	**0.0002**	0.762
**III**			/	**0.0002**	0.450
**IV**				/	**0.0002**
**V**					/

**Table 4:** Spearman’s correlation coefficients between protocols.

Correlations						
		I	II	III	IV	V
**Spearman’s rho**	**I**	**1.000**	0.770 **	0.648 **	**−0.042**	0.818 **
	**II**		**1.000**	0.939 **	**0.139**	0.988 **
	**III**			**1.000**	**0.091**	0.915 **
	**IV**				**1.000**	**0.152**
	**V**					**1.000**

** Correlation is significant at the 0.01 level.

## Data Availability

The original contributions presented in this study are included in this article.

## Supplementary Materials

The following supporting information can be downloaded at https://www.mdpi.com/article/10.3390/toxics13020113/s1. Figure S1. Flowchart of individual steps in each of five applied protocols.
