# Supplementary material for: The Influence of Different Protocols on the Application of the Dithiothreitol Assay in Determining the Oxidative Potential of Ambient Particles
Source: Toxics. 2025 Jan 30;13(2):113. doi: 10.3390/toxics13020113 (PMC11860352; doi:10.3390/toxics13020113)
Supplement: Supplementary file 1 [file toxics-13-00113-s001.zip › toxics-3447189-supplementary.pdf]

# The influence of different protocols on the application of the dithiothreitol assay in determining the oxidative potential of ambient particles

**Maja Jovanović[1]*, Marija Živković[1], Bojana Petrović[1], Saima Iram[2], Milena Jovašević-Stojanović[1], Svetlana Stevanović[2]**

## Supplementary material

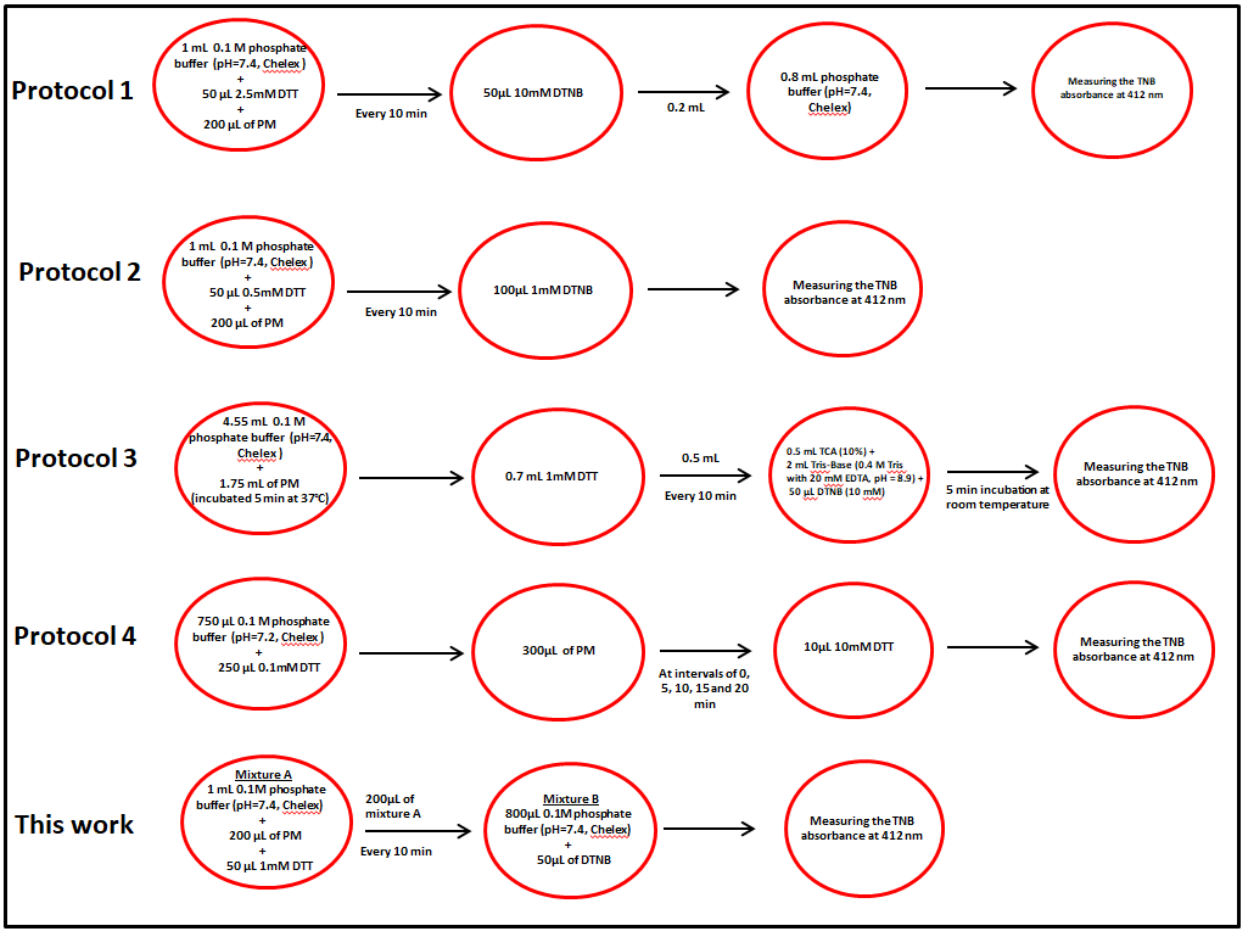


**Figure S1**. Flowchart of individual steps in each of five applied protocols.
